# Compact Electron Gun Based on Secondary Emission Through Ionic Bombardment

**DOI:** 10.3390/s110505202

**Published:** 2011-05-11

**Authors:** Babacar Diop, Jean Bonnet, Thomas Schmid, Ajmal Mohamed

**Affiliations:** Onera-The French Aerospace Lab, F-91761 Palaiseau, France; E-Mails: babacar.diop@onera.fr (B.D.); jean.bonnet@onera.fr (J.B.); thomas.schmid@onera.fr (T.S.)

**Keywords:** electron gun, electron beam fluorescence, secondary electrons, optical diagnostics, low density gas flow, density measurements, temperature measurements, 07.57.-c, 07.20.Dt, 33.50.Dq, 34.80.Dp, 34.80.Nz, 47.40.Ki, 52.25.Jm, 52.50.Dg, 61.80.Fe, 79.20.Hx

## Abstract

We present a new compact electron gun based on the secondary emission through ionic bombardment principle. The driving parameters to develop such a gun are to obtain a quite small electron gun for an in-flight instrument performing Electron Beam Fluorescence measurements (EBF) on board of a reentry vehicle in the upper atmosphere. These measurements are useful to characterize the gas flow around the vehicle in terms of gas chemical composition, temperatures and velocity of the flow which usually presents thermo-chemical non-equilibrium. Such an instrument can also be employed to characterize the upper atmosphere if placed on another carrier like a balloon. In ground facilities, it appears as a more practical tool to characterize flows in wind tunnel studies or as an alternative to complex electron guns in industrial processes requiring an electron beam. We describe in this paper the gun which has been developed as well as its different features which have been characterized in the laboratory.

## 1. Introduction

The electron gun [1,2] is the primary component of many instruments employed in industrial and monitoring processes (welding, lithography, physical vapor deposition, irradiation/sterilization, microwave RF devices, electron microscope, *etc.*). A less common application, but which motivates our research work in electron guns, is in the field of optical diagnostics of low density flows. This measurement technique, named Electron Beam Fluorescence (EBF) [3–5] exploits the fluorescence of atoms and molecules induced by an electron beam having a current of a few mA, an energy of about 20 keV and a diameter of a few mm propagating a few tens of cm in a low pressure (less than 100 Pa) gas. Of the different types of electron guns (thermionic [6], field or cold emission [7], photocathode [8], plasma cathode [9], ionic bombardment [10,11], *etc.*) which exist, up to now it has been more practical to use thermionic guns. In this most common gun, a heated cathode, built from massive tungsten or barium compound, emits low energy electrons which are then accelerated and focused on a target. This kind of electron gun has however several drawbacks for our intended in-flight application aiming at performing EBF measurements in the flow around an atmospheric reentry vehicle [12]. First, the heated cathode, the control electrode and the accelerating electrode must all be under secondary vacuum at a pressure lower than 10^−3^ Pa. Second, the power necessary for heating the cathode must be at least 20 W to produce an electron current of 1 mA and the power supply must be floated at the level of the accelerating voltage with the use of an isolation transformer. And third, the system is quite sensitive to mechanical vibrations and diaphragms must be finely aligned to maintain a good beam quality.

## 2. Secondary Emission through Ionic Bombardment Principle

**Figure 1.**
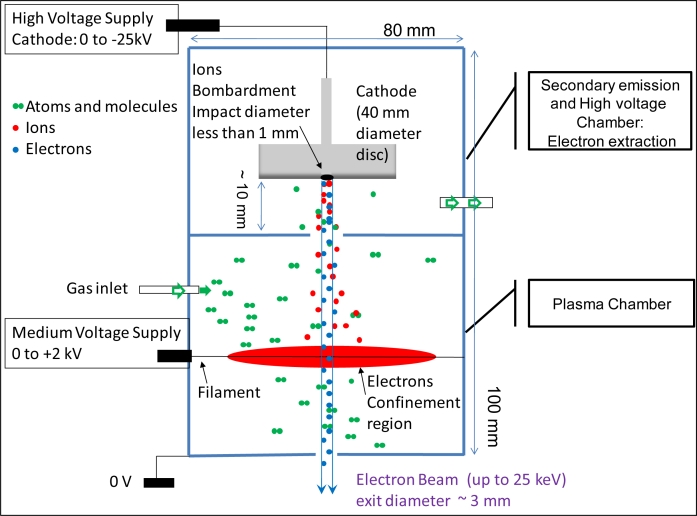
Electron beam emission principle.

The electron gun described here is based on the mechanism of secondary emission under ion bombardment [10,11] as depicted in Figure 1. Ions are produced in a plasma chamber at low pressure, about 2 to 8 Pa. To obtain a plasma at such pressures it is necessary to use electrostatic or magnetic confinement of electrons so as to dispose of a mean free path of electrons that is larger than the ion source dimensions. By electrostatic confinement the discharge is thus more easily created between a thin anode wire at high potential (200–800 V) and a surrounding cathode at ground potential [13]. Ions from this plasma are attracted through a small hole, 8 mm in diameter, toward the accelerating cathode at high potential (−25 keV in our case). They hit this massive cathode and secondary electrons are emitted during this process. The cathode is a 40 mm diameter disc made of aluminium alloy which has the best compromise between high electron emission efficiency [14] and low surface erosion through ionic bombardment. These electrons are then accelerated in the opposite direction and are also collimated by the electric field thereby producing the desired electron beam. The electron current measured at exit of the gun with a Faraday cup can be up to 1 mA with the power supplies described below and the adopted dimensions for the gun. The ion current is not directly measurable, due to the complex scheme leading to secondary emission. It is estimated, from the ratio of electron beam current over the power supply current, to be in the range 0.1 to 0.3 mA taking into account the secondary emission coefficient of aluminum [10] and some loss of electrons in the gun.

The electrons are emitted from a location on the cathode which can be assimilated as a point source about 300 μm in diameter. This emission occurs also with a very small divergence, making it easy to obtain a collimated beam 3 mm in diameter at gun exit. The anode wire is placed slightly off axis at about 1 cm from the fast electrons beam path and at this distance, the smaller electric field in the ion source seems to have no effect on the 25 keV electrons. The beam diameter is about 5 mm at 20 cm from the gun’s exit when the beam is controlled just by the geometry of the gun. A focusing coil can be used to reduce it to 1 mm if necessary. Neither a small diaphragm nor any precise adjustment of the beam position is required as in a thermionic emission gun. The beam current is controlled solely by the ion source current with a good stability if the internal pressure is constant. We have no measurement of the beam energy spectrum. We assume that all electrons are due to secondary emission at the cathode surface, and thus have the same energy. The low pressure in the gun leads to only a very few collisions which can reduce the energy. Most of the low energy electrons are filtered through dispersion by the electric field inside the gun or through blockage by the small diameter diaphragm at the exit of the gun. So we consider that the beam is essentially monoenergetic with an estimated dispersion of 100 eV due to causes like for instance power supply ripple.This mono-energetic property can be easily checked through an invariant beam diameter when a constant magnetic field is used for the deviation of the beam.

The main constraints for this gun come from the Paschen’s law. The plasma source needs a minimum gas pressure to ignite, about 2 Pa of air for the small dimensions of the plasma chamber in our case. But in the same time the accelerating cathode must not start a discharge, which imposes a maximum pressure of about 4 Pa and a gap of 1 cm between the cathode and the surrounding parts at ground potential. For this reason, the vacuum pump is connected to the cathode chamber in order to have the minimum pressure around the cathode. The electric field must stay below 30 kV/cm in the chamber in order to prevent vacuum arcing. There is no well defined limit for the low energy side. We found that it is possible to extract electron beam down to 1 kV, but the propagation range is then short (a few cm) and it is also quite difficult to obtain a sufficiently collimated beam for practical use.

The cathode is heated through the process of ionic bombardment. This heat represents 5 to 10 Watts to be evacuated through the insulator. The power dissipated in the ion source (anode wire) is about 6 W (15 mA at 400 V) which can also be easily evacuated through conduction from the gun body. The anode wire is at a much lower temperature than for a thermionic filament whose thickness and surface must also be of relatively small size (hairpin) for proper electron emission. The allowed dimensions and localization for the anode wire make it also insensitive to bombardments in the plasma. This component is thus much more resistant for long period and harsh vibration environment operation. The only consumable part of the gun is in fact the cathode disc which suffers erosion through the ionic bombardment. In our system, a small hole, 300 μm in diameter and 100 μm in depth, appears after about 20 hours of operation for 1 mA beam current operation. Such a hole has nearly no effect on the beam current, but increases the beam spreading. When the focusing device is no more able to compensate for this spreading, the cathode has to be changed or surface repolished.

Taking into account all these constraints and the experience acquired from previously constructed electron guns, a new compact system based on the above described principle has been designed for our aerospace application. The operating voltage is the result of a compromise between three effects concerning the wanted electron propagation range. This range increases with high voltage, but the required power increases also while gas excitation efficiency decreases following a ln(V)/ V law.

From our experience in wind tunnels we need a current of at least 1 mA in order to have an exploitable fluorescence signal from molecules in the low density conditions present at 60 to 80 km altitude zone. The compact differential pumping system which has been chosen allows efficiency (beam current over power supply current) of at most 40% for the electron gun. To approach this limit, one must then use a 25 kV power supply with a maximum current of 2.5 mA in order to ensure an electron beam current of 1 mA. With only minor adjustments of the operating conditions, pressure and ion source current, this electron gun can be used in various gases of interest for aerospace applications, such as air, nitrogen or carbon dioxide.

**Figure 2.**
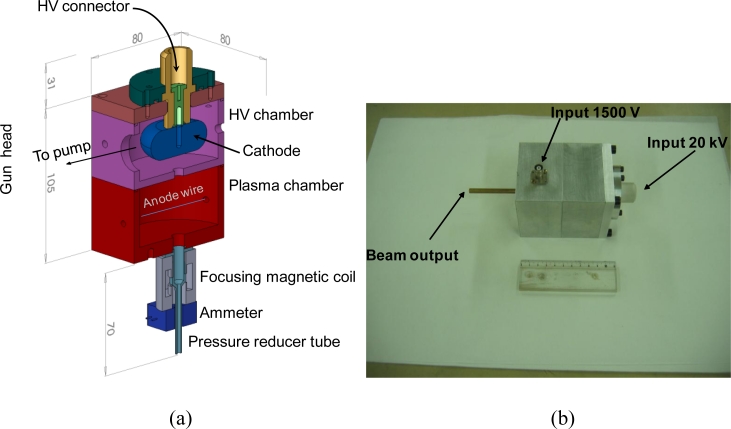
Electron gun head. **(a)** Electron gun head (all dimensions in mm). **(b)** Photography of the gun head (matched to a 110 mm long ruler).

Figure 2 presents a picture of the electron gun head prototype which has been manufactured for our study. It is of rectangular form with dimensions 80 mm × 80 mm × 146 with vacuum pumping directly on the high voltage chamber and is much more compact than all prototypes we have built up to now. A cylindrical form is more common but this rectangular form is more practical for mechanical coupling to the final instrument of our project.

## 3. Additional Features

We now present the additional features brought to this gun to ensure an exploitable electron beam for fluorescence measurements in a gas flow:

- Pressure reducer
- Beam focusing
- Beam pulsation
- Beam current measurement

### 3.1. Pressure Reducer

The static pressure of the gas or flow into which the beam is emitted will generally be higher than the internal pressure of the electron gun. In our fluorescence application this will range in between 10 and 100 Pa *versus* 2 to 8 Pa inside the gun. To ensure a smooth differential between these two pressures we can use a pressure reducer in the form of a metallic tube [Figure 2(a)] about 50 mm long and of internal diameter of a few mm. In our case, a diameter of 3 mm is found to be optimal in order to enable the propagation of a thin beam over a few hundred mm in outside pressures up to 100 Pa for a gun pressure around a few Pascal. In the situation where the gas to be probed is at a lower pressure, additional gas from a reservoir must be injected in the gun in order to ensure the adequate operational pressure in the ion source.

The reducer tube must be manufactured in an electrically conductive and non-magnetic alloy (Brass or stainless steel for example) to allow a clean straight propagation of the electron beam. This material must eventually sustain high temperatures for applications like in high enthalpy flow probing where gas will enter the pressure reducer at high temperature and, although it will be cooled partially through expansion and contact with the walls, temperature as high as 1,500 K can be reached on the tube. Simulation of the hot flow in the pressure reducer is complicated as the composition and excitation state of the incoming gas is poorly known, which can lead to large uncertainties and erroneous conclusions. The incoming flow is usually viscous and becomes molecular at the gun’s exit which further complicates the application of computational fluid dynamics codes. We therefore rely on experiments to test such a component and, fortunately, those made in high enthalpy wind tunnels [15] show no dramatic effect on the gun even for long runs (30 min) with a stainless steel pressure reducer.

### 3.2. Beam Focusing

Figure 2(a) also presents at the exit of the gun head a beam focusing magnetic coil system to further collimate the beam in the situation where the gas to be probed is at a relatively high pressure (close to 100 Pa). This component is placed on the pressure reducer tube described above. Usually a coil allowing a magnetic field of intensity less than 500 Gauss is sufficient to correct the dispersion of a 20 keV electron beam into a reasonable diameter of a few mm for a propagation length of about half a meter. A simple solution for this beam focusing system is to use the magnetic coil of a commercial cylindrical electromagnet like the Mecalectro model 8.10.BA.83. This element operates at low voltage (24 V) and needs only a low current (up to 0.2 A) for a maximum power consumption of 8.5 W, which is quite interesting for an in-flight operation. The other parameters for the Mecalectro coil are the following: it is of cylindrical form of length equal to 40 mm, of outer diameter equal to 30 mm, of inner diameter equal to 10 mm and of weight equal to 140 g.

### 3.3. Beam Pulsation

Beam pulsation with a square function structure and half duty cycle at a frequency near to 10 Hz is required in our application in order to monitor the luminous background when the electron beam is off. This frequency order value is set by the data acquisition and sensitivity possibilities of a commercial miniature spectrometer (HR4000 model from the Ocean Optics, Dunedin, FL, USA) we intend to use to acquire spectra with comfortable signal to noise ratio in typical EBF experiments. Our other requirements are that the main properties of the beam (nominal current, energy and geometry) must be maintained for at least 90% of the pulse duration and for the time period when the beam is switched off, the residual electron current should be less than 1% of its nominal value in order to acquire an acceptable background signal.

Different solutions can be sought to ensure the pulsation of an electron beam. We have essentially evaluated the following two options:

1. Switching on and off either the high voltage or the discharge : but this solution provides a very unstable electron beam which does not satisfy the requirements listed above for the target frequency of 10 Hz.
2. Deviation of the beam off the gun’s exit with the help of an electric or magnetic field.

**Figure 3.**
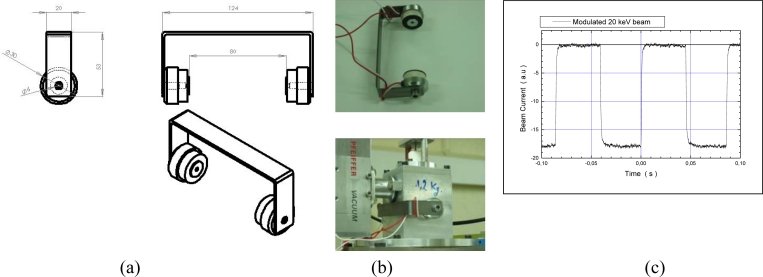
Components and positioning of the magnetic coils for the electron beam deviation system. **(a)** Design of the magnetic deviation component. **(b)** Set up on the electron gun. **(c)** Example of results obtained for the intensity of the beam pulsed with the magnetic coils.

Deviation by an electric field is not usable inside the gun, where electrons ionize the ambient gas and produce a discharge between electrodes, as this electric field will be modified by the plasma causing instabilities to a required deviation. Such a deviation scheme can be used on the pressure reducer tube, but the electron beam blocked inside the tube will cause rapid heating of the tube and in certain cases, instabilities due to charge accumulation in the tube. The best solution resides in the magnetic deviation principle with two coils placed outside the gun but very close to the plasma chamber. A field intensity of 10 Gauss in the region between the cathode and the ion source (at the same position of the diaphragm between these two compartments of the gun) is found to be sufficient for deviation of the beam by about 2 mm from the diaphragm centre. This in turn is sufficient to sweep the beam away from the 3 mm diameter input of the pressure reducer. Two magnetic coils (Mecalectro magnetic clutch 5.80.01), operated with a current less than 200 mA at 12 V and at maximum power consumption of 2 Watts has been implanted on our prototype as depicted in Figure 3.

The weight of the coils and the associated mechanical support amounts to about 200 g whereas the electronic module to provide the pulsed square function from a continuous electrical current and of size 90 × 40 × 40 mm is also of moderate weight (90 g). This deviation module therefore increases only slightly the global weight and dimensions of the whole gun assembly.

Figure 3(c) presents the measured intensity of the electron beam when the current in the magnetic coil is pulsed with a square function at 11.5 Hz. The intensity is verified to be oscillating from zero to its maximum value (1 mA in this experiment) with a rise time of 5 ms, limited by the self-inductance of the coils, which accounts to less than 10% of the plateau portion of the pulse. The intensity in the plateau region of the pulse is relatively stable at 3% of the nominal value and reproducible from pulse to pulse up to a frequency of 50 Hz beyond which the plateau region disappears in the pulses.

### 3.4. Beam Current Measurement

Measurement of the electron beam current intensity is needed to normalize the density measurements to be performed on fluorescence intensity acquisitions. Usually in ground facilities one uses a Faraday cup placed at a few hundred mm from the gun’s exit. However, we cannot rely on such a solution for an in-flight application where all detectors must be placed aside as the electrons emitter. The most promising way is to use the Hall effect of a magnetic coil around the electron beam [Figure 2(a)]. The most miniature commercial devices which are available are for relatively high current, usually higher than several tens of mA. We complemented one such commercial device, the model LA55 from the Lem company, with a low noise current amplifier and succeeded to measure currents of the order of 1 mA.

**Figure 4.**
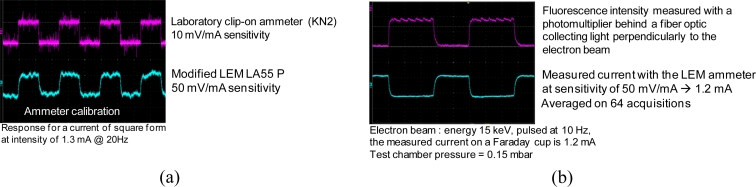
Electron beam current measurements with the amplified LEM LA55P ammeter. **(a)** Calibration of the beam current with a standard laboratory ammeter (KN2). **(b)** Measurement of an electron beam current in a vacuum chamber.

Figure 4(a) presents some results of the calibration of the LEM device compared to a standard laboratory low-current clamp-on ammeter (KN2 from the Chauvin-Arnoux company). Although the LEM signal appears noisier, these tests validated that the device has the required sensitivity for measuring the low electron intensity of our EBF assembly. This is confirmed in Figure 4(b) where a measurement of a current of 1.2 mA (checked with a Faraday cup) for the electron beam is presented. The measurement is also matched to the measurement of the visible fluorescence intensity performed with a fiber optic coupled to a high-bandwidth photomultiplier placed outside the vacuum chamber. Both curves exhibit the same rising and falling times which confirms that the LEM device has the necessary time bandwidth for our application. The measurement shown is averaged over 64 acquisitions but in high speed probing conditions such averaging will not be allowed. Therefore, as a future improvement, we need to reduce the noise through a better shielding of the LEM device against magnetic noise (particularly from the magnetic field of the power supply used for pulsing the beam) and through optimization of the current amplification system.

## 4. Integration of the Electron Gun into an EBF Instrument

**Figure 5.**
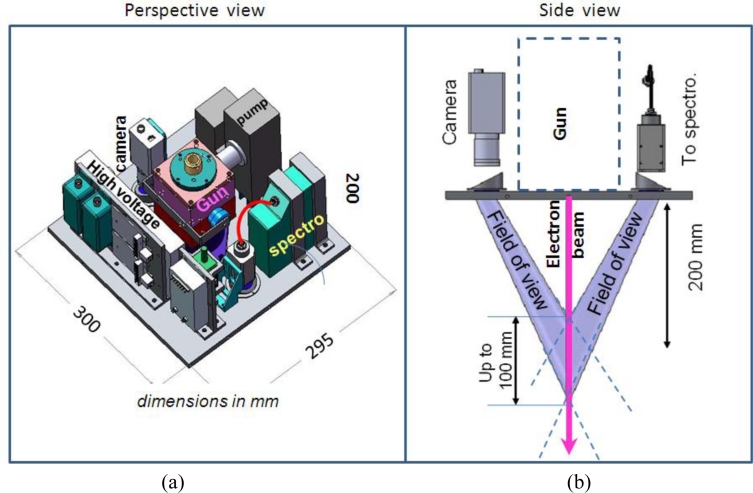
**(a)** Layout of the Electron Beam Fluorescence integrated instrument. **(b)** Optical detectors configuration.

As an example of application of the electron gun developed and described above, we present in this chapter its integration in a portable set up (Figure 5) aiming at performing electron beam fluorescence measurements in a static gas or in a flow. The set up encompasses the following elements in addition to the gun:

- a Turbomolecular pump (model referenced TPD 011 from the Pfeiffer company) to enable the 2 to 8 Pa pressure regime in the gun.
- a moderately high voltage power supply to create the plasma in the gun. The model ‘2A24-N30-M’ from the ULTRAVOLT company has been chosen as it can deliver a high voltage up to 2 kV with a current of 15 mA with operation from a 24 V source for a power consumption of 30 W. It has also the capacity to sustain high mechanical vibration levels up to 10 g (shock up to 20 g) while together being of small dimensions and low weight (1.18 kg).
- a high voltage power supply to provide the required potential to enable an electron beam of up to 25 keV energy. Here also a model from the Ultravolt company has been chosen (25C24N60, 25 keV, 2.4 mA, 60 W). It is also of modest dimensions, weights 300 g and has the same operational voltage (24 V) and environmental resistance as the 2 kV unit.
- pressure gauge: pressure measurement inside the gun head is needed to check if pressure is low enough (a few Pa) in order to switch on the high voltage power supply (else electrical arcing is feared to destroy the power supply or elements inside the gun). The choice converges to the model ‘Leybold THERMOVAC TTR 91’ which is one of the most miniature absolute pressure gauges on the market and offering environmental resistance compatible to the flight operation capacity for our instrument.

For the characterization of induced fluorescence, two optical detectors are used:

- a small size CCD camera to monitor the spatial properties of the beam as well as to perform density measurements in a gas. The model scA640 model from the Basler company is presently incorporated in our setup. It has 658 × 492 pixels with maximum acquisition speed of 74 frames per second for 8 bit intensity resolution (which can be increased to 16 bits if pixels are grouped and acquisition rate diminished)
- a miniature spectrometer (HR4000 model from Ocean Optics) to analyze spectrally, between 200 to 500 nm sampled with a 4,000 pixels array detector, the contents of the fluorescence at a chosen spatial point (about 200 mm from the gun’s exit in our case) for gas chemical identification as well as temperature measurements.

Miniature optical objectives and sapphire windows in the form of prisms (apex angle of 30°) are implemented in the setup in front of these detectors to monitor the fluorescence of the beam up to a distance of 300 mm from the gun exit. All these components hold in a box roughly of 300 × 300 × 250 mm dimensions and the total weight of the system is around 11 kg. The overall power consumption is less than 130 W : 60 W for the cathode power supply, 30 W for the ion source, 24 W for the turbopump, 1.2 W for the focusing coil (12 V–100 mA), nearly 2 W for the beam pulsation system and a few Watts for the optical detectors (CCD camera and spectrometer) and control electronics. These features are compatible with the requirements for in-flight experiments onboard a vehicle such as described in Reference 12 or onboard a rocket for characterizing locally the atmosphere at high altitudes between 50 and 100 km [16]. Detailed results of the experimental validation of this set up in a wind tunnel will be published soon. We present below only a few results of the setup operated in a static gas.

## 5. Tests of a Complete EBF System in a Static Gas Cell

**Figure 6.**
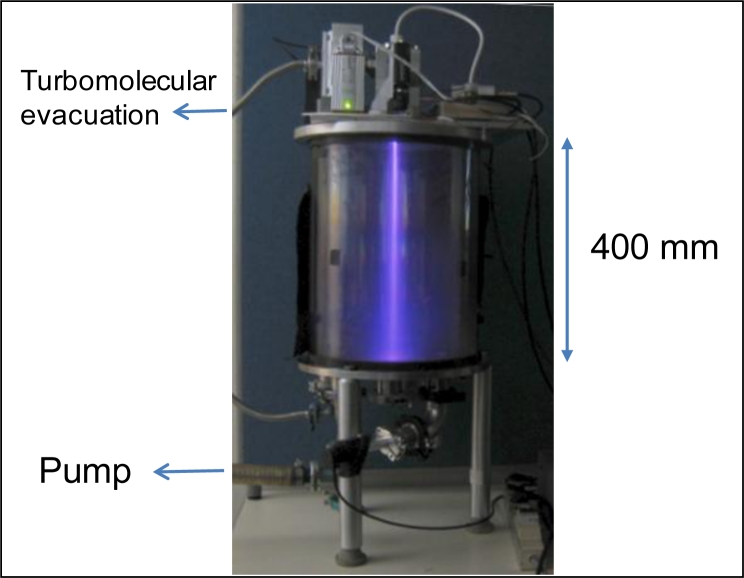
Emission of a beam in the transparent vacuum chamber.

The validation tests of the prototype we built have been performed in a small transparent cylindrical vacuum chamber of diameter 300 mm and of length 400 mm with the cylindrical part made of optical glass having a thickness of 8 mm. There are metallic plates at top and bottom of this cylinder. The EBF assembly base plate has been adapted to the top plate of the cylinder where appropriate holes have been drilled for the prism windows and electron beam exit (Figure 6). The bottom plate is equipped with all necessary feedthroughs for pumping and pressure monitoring. The EBF setup can be controlled manually with an electronics box (19 inch 2U rack format) regrouping all the control units for the different commercial components as well as a converter to provide 24 V power supply from wall-plug 220 V power supply. All the functions of this electronic box can also be obtained through an electronic card implemented in the assembly and which can be remotely controlled by a computer through the USB protocol.

**Figure 7.**
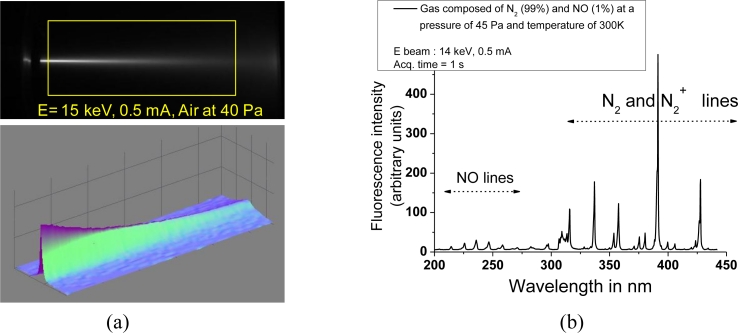
Examples of typical acquisitions obtained with the EBF system. **(a)** CCD image (top) of the fluorescent beam in air gas with typical dispersion and attenuation shown in the 3-D trace (bottom). **(b)** EBF spectrum in a gas mixture of N_2_ + NO.

In Figure 6, we can visualize the fluorescence trace of the electron beam in the cylinder at a pressure of 30 Pa. The high voltage is limited to 15 keV to avoid X-ray emission crossing the glass wall of the cylinder. Such operation can be maintained for at least one hour before excessive heating of the components. A longer operation time is possible with simple air or water cooling schemes. Figure 7 illustrates typical acquisitions which are obtained with the optical detectors of the set up:

- image mapping of the fluorescent beam from which local density can be derived after proper correction of beam dispersion and attenuation as depicted in the 3D view presented in the same figure
- spectrum of the fluorescence excited all along the beam showing molecular nitrogen and nitric oxide lines

Analysis of such data in a static gas or in flows of low density wind tunnels will be published soon.

## 6. Conclusions

A new compact electron gun based on the secondary emission through ionic bombardment principle has been developed and integrated in a compact EBF assembly. All the main key components (electron gun, turbo pump, high voltage converters, camera and spectrometer) fit in a box less than 300 mm × 300 mm × 250 mm and weighting about 11 kg, with only a few electrical interfaces. The laboratory prototype presented has been validated to emit a well collimated electron beam of energy up to 25 keV energy and current up to 1 mA current which propagates over a distance of at least 300 mm in a gas at pressures up to 100 Pa and provides reference images and spectra of the fluorescence induced in the gas along the beam. All these features are in conformance to the specifications of an in-flight experiment in the high atmosphere which motivated this study. The testing of the whole setup in the vacuum conditions of a wind tunnel (Mach 2 MARHy in Orléans, France) has been recently performed successfully and the detailed results as well as the flow measurements acquired will be published soon. Most of the components have also a potential to sustain mechanical vibration and shock up to 20 g. The complete system can be used in different gases, like air, CO_2_, helium, *etc*. or in a composition of these gases. In fact, with small adjustments of pressure and operating voltages the electron gun can be used in any kind of gases, even corrosives ones. The remarkable performance features of the gun developed can be advantageously used in other fields where a robust and yet easy to operate electron beam is needed, like in welding, CVD deposition *etc.*
